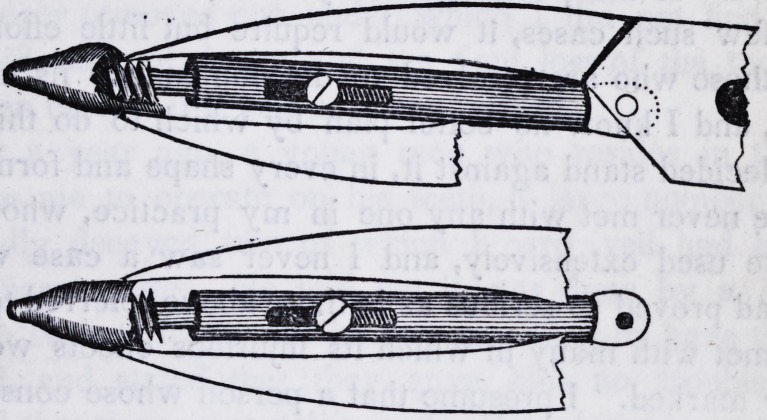# Compound Root Forceps

**Published:** 1844-06

**Authors:** S. P. Hullihen

**Affiliations:** Wheeling, Va.


### ARTICLE IV.

*Compound Root Forceps.* By DR. S. P. HULLIHEN, Wheeling, Va.

THE above named forceps were contrived some time since, for the purpose of extracting hollow roots of teeth, with more expedition and at the same time, with less pain to the patient than was possible with the instruments in general use, and as the forceps have fully answered the purpose for which they were intended, I have thought them of sufficient importance to lay them before the profession.

The Compound Root Forceps are about nine inches in length, and like the common straight forceps with the exception that the beak is much longer, and much narrower and thinner at the point. Lengthwise, within and between the blades of the beak is a steel tube, one end of which is open; the other solid and flat and jointed in a mortise in the male part of the forcep's joint. When the forceps are opened, this joint permits the tube to fall backwards and forwards from one blade of the beak to the other, without any lateral motion. Within this tube is a spiral spring which forces up a shaft—two thirds of the length of the shaft is rounded and fitted neatly into the tube, the other part is a well tapered or conical screw. The shaft is retained in the tube by a small screw, that is fixed into the shaft through a notch half an inch long in one side of the tube. The shaft and tube are so fitted together, and to the beak of the forceps, that one half of the rounded part of the shaft projects beyond the end of the tube; so that the shaft may play up and down upon the spring the length of the notch, and the screw part projecting beyond the point of the forceps, so that the shaft may be embraced between its blades, just behind the base of the screw. A full sided view of the beak of the forceps with its tube and shaft is well represented in the several cuts.

The forceps are used, by first embracing the shaft between the blades. Then screwing it gently and as deeply into the root as possible, the blades are opened—pushed up upon the root, which is then seized in either of the ways as the case may require, represented in the annexed cuts.

The screw thus combined with the forceps, prevents the root from being crushed. It acts as a powerful lever when a lateral motion is given; it is likewise of advantage when a rotary motion is made—it prevents the forceps from slipping, or of their action being lost, should even one side of the root give way in the act of extracting it—and is used with equal advantage where one side of the root is entirely gone. In short, this combination of the screw and forceps forms an instrument which fulfils every indication that can be desired in the extraction of hollow roots.

The shaft of the Compound Root Forceps, is easily changed; a number of different sized screws may therefore be used in the same pair of forceps.

## Figures and Tables

**Figure f1:**
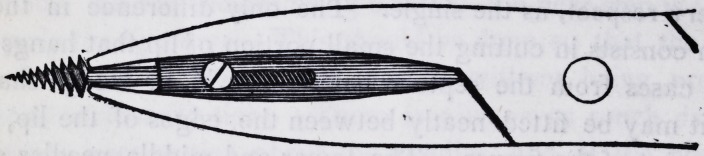


**Figure f2:**